# Natural medicines target tumor vascular microenvironment to inhibit tumor

**DOI:** 10.1016/j.gendis.2025.101623

**Published:** 2025-04-08

**Authors:** Yirui Lu, Zhiliang Guo, Hong Li, Jiao Wen, Xiaoyun Zhang, Xiumei Guan, Xiaodong Cui, Min Cheng

**Affiliations:** a School of Basic Medicine Sciences, Shandong Second Medical University, Weifang, Shandong 261053, China; b The 80th Group Army Hospital of Chinese PLA, Weifang, Shandong 261021, China

**Keywords:** Angiogenic factors, Natural medicine, Tumor, Tumor microenvironment, Vascular microenvironment

## Abstract

The abnormally active tumor vasculature provides a good blood supply for the rapid proliferation of tumors. The tumor microenvironment of tumor cells is associated with the secretion of a lot of angiogenic factors to promote the formation of blood vessels. However, the blood vessels are often irregular and immature. Additionally, the tumor tissue, in the process of its rapid proliferation, oppresses tumor blood vessels, causing hypoperfusion and leading to high interstitial pressure and hypoxia, which also results in changes to the fluid mechanics in the tumor microenvironment. Different fluid mechanics affect circulating tumor cell behavior and control various functions. A good mechanical microenvironment may be one of the important targets for inhibiting tumor proliferation and migration. Therefore, regulating tumor blood vessels to maintain a steady fluid mechanical microenvironment has the potential to be one of the key targets for tumor treatment. Numerous studies have demonstrated that certain natural medicines exhibit significant potential for inhibiting tumor growth and metastasis by selectively targeting tumor blood vessels, regulating the production of angiogenic cytokines, facilitating vascular normalization, *etc*. Furthermore, natural medicines enhance the anti-tumor effects of chemoradiotherapy and act as adjuvant agents to alleviate its associated side effects. This review summarizes the angiogenesis of the tumor microenvironment, changes induced by mechanical conditions, and the response of tumor cells and vasculature to different fluid shear stress to promote vascular normalization treatment strategies.

## Introduction

Since the 1970s, targeting the tumor vasculature system has become a pivotal focus in cancer treatment.^1^^,^^2^ Anti-angiogenic therapies reduce tumor nutrient supply and inhibit tumor growth. Simultaneously, maintaining a healthy and effective blood supply is essential for stabilizing the tumor vascular microenvironment and ensuring the delivery of chemotherapy drugs to tumors. Therefore, promoting tumor vascular normalization is also a crucial strategy in anti-cancer treatment.^3^

Tumor microenvironment (TME) is a complex milieu characterized by the presence of tumor-associated immune cells, stromal cells, vasculature, cytokines,^4^ and exosomes.^5^ The vasculature plays a pivotal role in the tumor proliferation and metastasis.^2^ Within TME, hypoxia instigates cancer cells to secrete vascular endothelial growth factor A (VEGFA) leading to the development of irregular, non-uniform, and immature blood vessels. Those blood vessels result in an inadequate blood supply and vascular leakage, thereby fostering the dissemination and metastasis of tumors.^6^ At the same time, the growth of tumor oppresses capillaries and lymphatic vessels. In this process, the deformation and permeability changes, and tumor blood vessels will worsen the interstitial pressures within the tumor tissue.^7^ Meanwhile, changes in vascular geometry lead to reduced fluid shear stress (FSS) within tumor capillary buds. In environments of high mechanical shear forces, circulating tumor cells (CTCs) experience increased mechanical stress, inducing their fragmentation and death, whereas, under lower blood shear forces, CTCs' activity increases.^8^ Additionally, increased permeability of tumor vasculature raises interstitial fluid pressure, leading to tissue hypoxia, vascular remodeling, and elevated interstitial pressure. On one hand, CTCs traverse larger endothelial gaps, accelerating tumor extravasation and secondary tumor formation. Moreover, elevated interstitial fluid pressure facilitates the diffusion of tumor cells, pro-tumorigenic factors, and extracellular vesicles from lymphatic vessels, significantly enhancing tumor invasion and metastatic potential.^9^

Furthermore, numerous natural compounds regulate key molecules involved in abnormal tumor angiogenesis, thereby inhibiting immature vessel formation and promoting vascular normalization.^10^, ^11^, ^12^, ^13^, ^14^ Thus, inhibiting pathological angiogenesis and promoting the normalization of tumor vascular morphology and function could maintain vascular microenvironment homeostasis, potentially serving as critical targets for combating tumor metastasis and enhancing anti-cancer drug delivery.

## TME and tumor vasculature

### The constituents modulate the vasculature within TME

#### Immune cells

**Figure 1:**
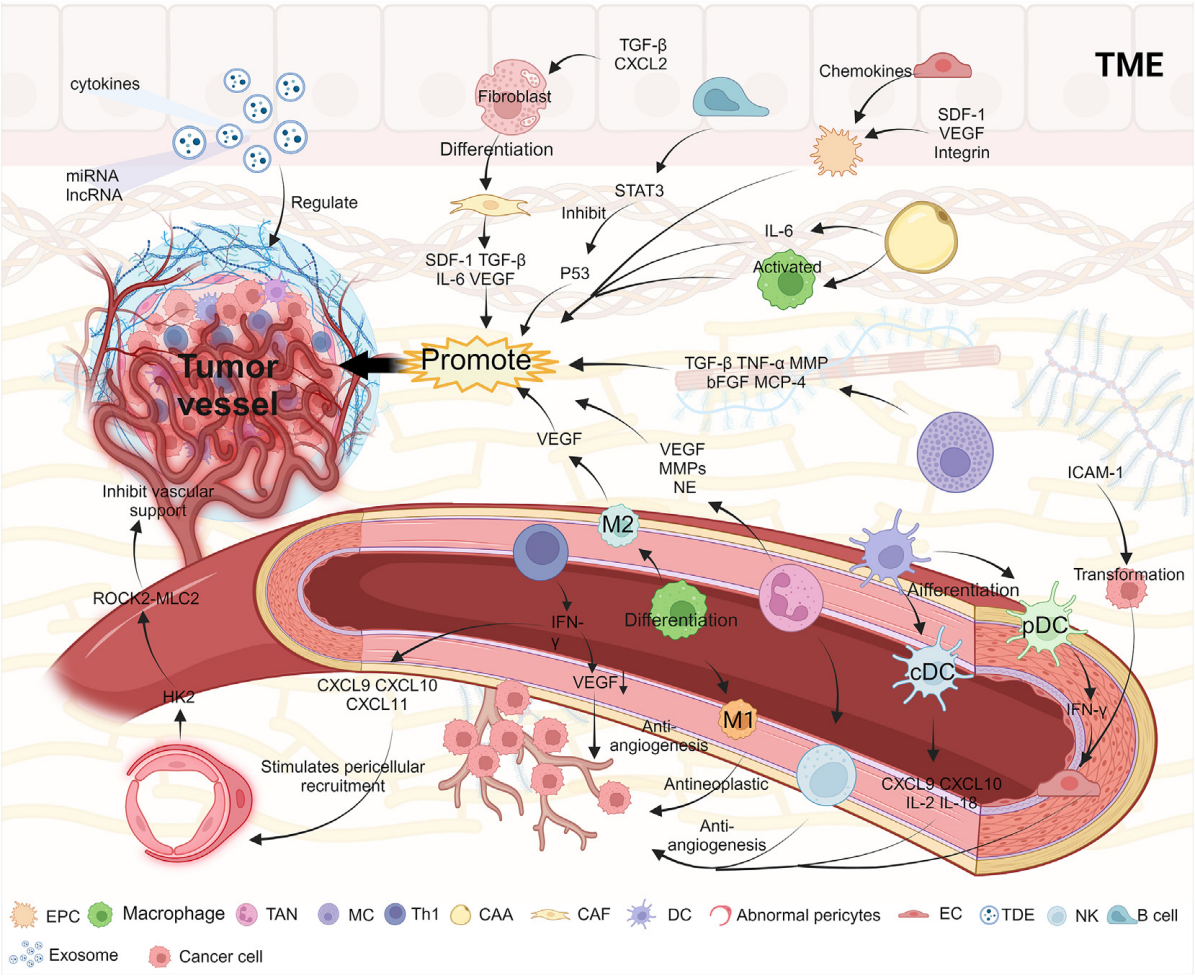
TME and tumor blood vessels. A diverse array of cellular and non-cellular components within TME intricately modulates various angiogenic factors. Certain cells exhibit either pro-tumor angiogenic or anti-tumor angiogenic effects under different stimuli, leading to either the expansion or regression of the tumor. TME, tumor microenvironment; EPC, endothelial progenitor cell; TAN, tumor-associated neutrophil; MC, mast cell; CAA, cancer-associated adipocyte; CAF, cancer-associated fibroblast; DC, dendritic cell; EC, endothelial cell; cDC, conventional dendritic cell; pDC, plasmacytoid dendritic cell.

Immune cells within TME play a pivotal role in the modulation of tumor vasculature function. Tumor-associated neutrophils (TANs), a predominant subset of white blood cells, were originally attributed a defensive role against malignancies. Nonetheless, mounting evidence suggests that TANs could indeed foster the proliferation, infiltration, angiogenesis, and metastasis of diverse tumor varieties.^15^ Studies have demonstrated that TANs possess the capability to induce angiogenesis in TME through the release of VEGF and other angiogenic factors.^16^ Additionally, TANs secrete matrix metalloproteinase (MMP8/9) and neutrophil elastase (NE) to modulate the extracellular matrix, thereby further facilitating angiogenesis (Fig. 1).^17^ Natural killer (NK) cells exert a dual role in angiogenesis, which is associated with the crosstalk between NK cells and neutrophils. On one hand, interleukin (IL)-12 produced by TANs induces NK cells to generate C-X-C motif chemokine 10 (CXCL10), perforin, and granulocyte enzymes, which show anti-angiogenic effects. On the other hand, NK cells produce interferon-gamma (IFN-γ) and granulocyte macrophage-colony stimulating factor (GM-CSF), inducing TANs to release VEGF and MMP9, promoting angiogenesis (Fig. 1).^18^ The density of tumor-associated macrophages (TAMs) typically correlates with the vascular density within the tumor tissue. TAMs are categorically divided into two distinct phenotypic groups, commonly referred to as M1 and M2.^19^ Within TME, M1 macrophages play a pivotal role in enhancing the tumor immune response and exerting anti-tumor effects. Conversely, M2 macrophages indirectly facilitate tumor progression by promoting angiogenesis and suppressing the function of cytotoxic T cells.^20^ Notably, M2 macrophages secrete VEGF, which not only contributes to tumor angiogenesis but also reinforces the M2 phenotype (Fig. 1).^21^ In addition, hypoxia-inducible factor-1 alpha (HIF-1α) inhibitors can organize TAMs to M2 polarization, but send M1 polarization, promote its inhibiting tumor angiogenesis, and promote its maturity.^22^ B cells activate the transcriptional activation factor 3 (STAT3), subsequently inhibiting the expression of P53 (Fig. 1). This inhibition leads to increased expression of angiogenic factors within the tumor.^23^ An investigation by Kam et al revealed that cancer-derived high mobility group protein B1 (HMGB1) stimulated angiogenesis by modulating B cell proliferation in esophageal squamous cell carcinoma.^24^ Mast cells are capable of secreting various factors, including transforming growth factor β (TGF-β), tumor necrosis factor-alpha (TNF-α), MMPs, and basic fibroblast growth factor (b-FGF). Among these, mast cell-specific serine protease, monocyte chemotactic protein-4 (MCP-4), is released in capillaries and epithelial basement membranes, activating pro-MMPs, thereby promoting angiogenesis (Fig. 1).^25^ Mast cell tryptase also plays a significant role in angiogenesis by promoting the proliferation of endothelial cells (ECs) and degrading connective tissue stroma, thereby creating a conducive environment for the development of new blood vessels.^26^ While some tumor-associated immune cells contribute to the disarray of tumor vasculature, Th1 cells have a positive impact on the functionality of tumor vasculature. IFN-γ, a characteristic factor produced by Th1 cells, plays a critical role. IFN-γ reduces the expression of VEGFA in ECs and increases the production of chemokines, such as CXCL9, CXCL10, and CXCL11 (Fig. 1). This, in turn, promotes pericyte recruitment, enhances vascular permeability, improves perfusion efficiency, and alleviates tumor hypoxia.^27^ Dendritic cells exhibit regulatory behavior based on their level of maturation in the context of tumor angiogenesis. Mature dendritic cells are categorized into two subtypes: conventional and plasmacytoid dendritic cells.^28^ The former exerts inhibitory effects on tumor angiogenesis by releasing angiogenesis-suppressing chemokines (such as CXCL9 and CXCL10) as well as anti-angiogenic factors (including interleukin (IL)-2 and IL-18). Similarly, the latter inhibits tumor angiogenesis through the secretion of IFN-γ, which hinders EC proliferation and motility by modulating toll-like receptor 7 (TLR7) signaling (Fig. 1).^29^^,^^30^

#### Stroma cells

Several crucial factors controlling tumor angiogenesis are synthesized by stromal cells within TME. Cancer-associated adipocytes and fibroblasts secrete paracrine signals such as stromal-derived factor-1 (SDF-1), TGF-β, IL-6, and VEGF (Fig. 1). These factors play a significant role in regulating the formation of EC tubules within TME and promoting the transition of ECs into a mesenchymal phenotype.^31^ Cancer-associated adipocytes exhibited fibrosis and pronounced vascularization, accompanied by elevated expression levels of IL-6, CXCL1, monocyte chemotactic protein-1 (MCP-1), CXCL2, and matrix metalloproteinase inhibitor 1 (TIMP-1).^32^ Hypoxia or the condition of low oxygen tension triggers the up-regulation of HIFs. In cancer-associated fibroblasts, the increased expression of HIF-1α can amplify vascular functionality and bolster blood perfusion, ultimately contributing to elevated tumor progression.^33^ Cancer-associated adipocytes stimulate EC proliferation and capillary formation, thereby facilitating the growth of highly vascularized tumors. This process was accompanied by an up-regulation of IL-6, CXCL1, MCP-1, MIP-2, and tissue inhibitor of TIMP-1.^32^ Hepatic stellate cells are mesenchymal-origin stromal cells found in the liver. Upon activation, they up-regulate the expression of cysteine-rich 61 (Cyr61) and subsequently induce the expression of growth factors, including VEGF. This up-regulation of VEGF by activated hepatic stellate cells promotes tumor angiogenesis.^34^

### Exosomes

Exosomes are small lipid bilayer vesicles ranging in size from 40 to 160 nm. They serve as essential vehicles for cell communication within TME.^35^ Tumor-derived exosomes and their contents actively participate in the establishment of tumor vasculature (Fig. 1).^36^

**Figure 2:**
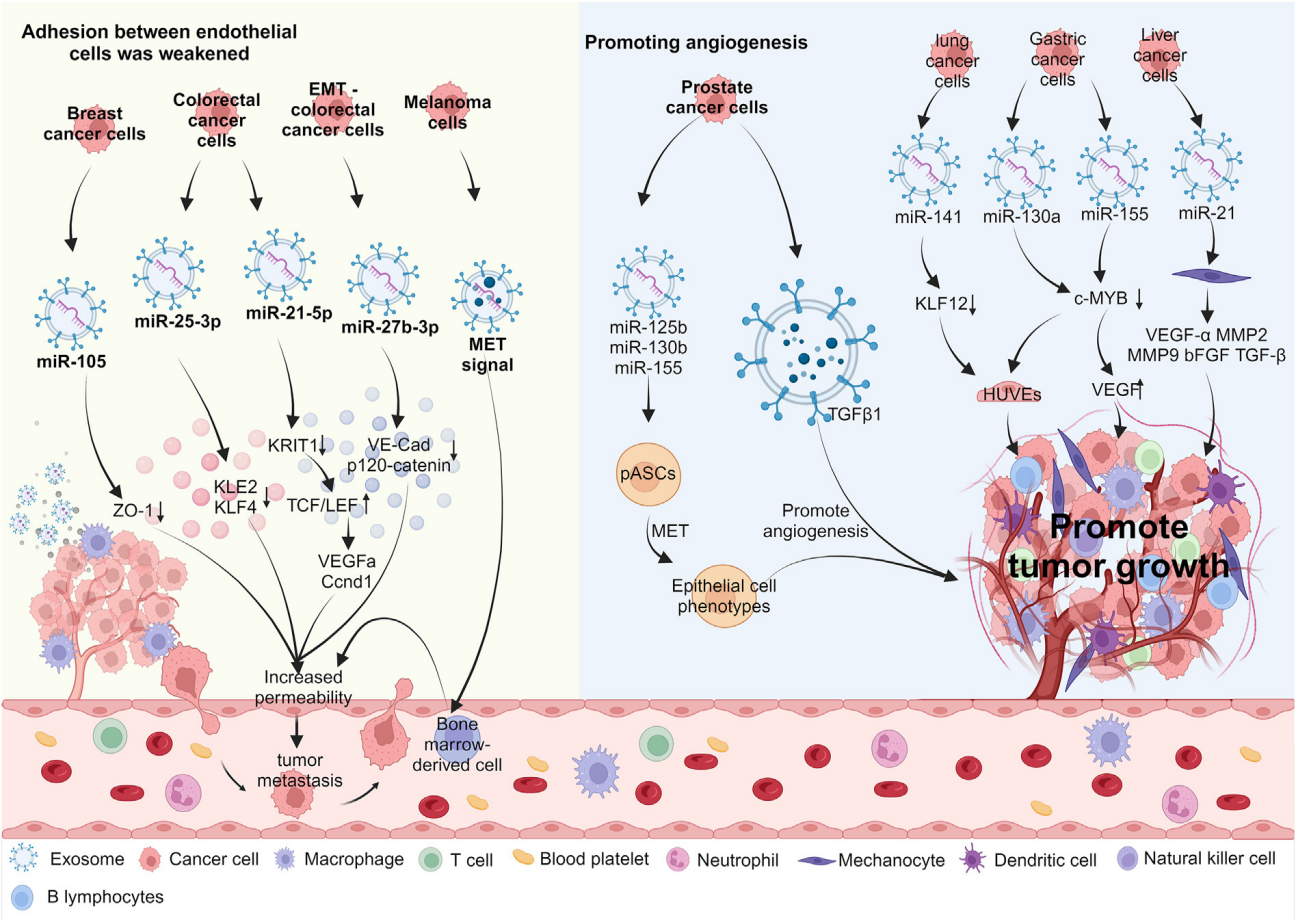
Tumor blood vessels are regulated by tumor-associated exosomes. Exosomes released by diverse cancer cells facilitate the transport of miRNAs, proteins, *etc*. They intricately modulate vascular permeability within the tumor microenvironment, thereby contributing to the evasion and metastasis of tumor cells. Moreover, exosomes originating from the tumor play a pivotal role in fostering tumor growth. MET, epithelial transition factor; pASCs, adipose-derived stem cells; VEGFa, vascular endothelial growth factor a; c-MYB, c-MYB transcription factors; HUVECs, human umbilical vein endothelial cells.

Tumor-derived exosomes can transport pro-angiogenic cytokines within glioblastoma, including VEGF, FGF, TIMP-1/2, IL-8, and IL-6, thereby instigating both angiogenesis and glioblastoma cell proliferation.^37^ Moreover, they influence tumor invasion and metastasis through the modulation of tight and adherent junctions of ECs. Specifically, exosomal miR-105 induces the down-regulation of tight junction protein, commonly referred to as zonula occludin 1 (ZO-1), thereby impairing the barrier function of the endothelial monolayer (Fig. 2). This perturbation heightens vascular permeability, consequently promotes the *in vivo* metastasis of cancer cells.^38^ Exosomal miR-23a released by lung cancer cells also targets ZO-1 in human umbilical vein endothelial cells (HUVECs). This interaction increases vascular permeability and enhances the migration of cancer cells.^39^ Exosomal miR-25-3p derived from colorectal cancer cells is transferred to the vascular endothelium and then promotes EC migration by silencing KLF transcription factor 2/4 (KLE2/4) (Fig. 2).^5^ In melanoma, tumor-derived exosomes exert the effects on bone marrow-derived cells through the mesenchymal–epithelial transition factor (MET) signaling (Fig. 2). This signaling pathway promotes angiogenesis and vascular permeability, ultimately leading to an increase in tumor invasion and metastasis.^40^

Prostate cancer cells secrete exosomes which play a crucial role in driving the MET of porcine adipose-derived stem cells. These exosomes carry specific miRNAs, such as miR-125b, miR-130b, and miR-155, which actively participate in the formation of tumor blood vessels (Fig. 2).^41^ Moreover, exosome TGFβ1 plays a pivotal role in stimulating angiogenesis in prostate cancer. Hence, exosomes emerge as indispensable elements in the facilitation of tumor angiogenesis (Fig. 2).^42^

### Tumor-associated endothelial cells

Within TME, persistent pro-angiogenic signals contribute to the proliferation of ECs. This manifests as vascular malformation and heterogeneity in vessel diameter, resulting in suboptimal perfusion within the tumor and elevated interstitial fluid pressure. Concurrently, tumor blood vessels exhibit characteristics such as inadequate binding between pericytes and ECs and an uneven distribution of pericytes. These features collectively contribute to heightened permeability and increased vascular fragility.^6^ Compared with the normal development of blood vessels, the blood perfusion of the tumor vasculature efficiency declines, leading to a TME hypoxia and the change of FSS.^43^ HIF-1 is induced by hypoxia and expressed highly in tumor tissues, associated with the migration and invasion of tumors. HIF-1 also promotes the expression of VEGF and MMPs and enhances the capacity of the EC migration and invasion, thus promoting angiogenesis.^44^ Meanwhile, in glioblastoma, HIF-1α can increase periostin (POSTN) transcription, promote TANK-binding kinase 1 (TBK1) phosphorylation in ECs, and enhance EC migration and tube formation.^45^ In addition to the abnormal increase of vascular growth factors, pathological shear force and abnormal increase of interstitial flow also control the formation of tumor blood vessels. For example, shear stress in the physiological state, capable of attenuating VEGF-induced angiogenesis, attenuated the invasive capacity of ECs. The interstitial fluid flow, on the other hand, is to promote the budding of EC invasion and blood vessels.^46^ Therefore, maintaining a healthy blood vessel microenvironment may reduce the abnormal proliferation of tumor ECs.

Endothelial progenitor cells (EPCs) assume a pivotal role in tumor angiogenesis.^47^ The enhanced secretion of VEGF and SDF-1 within tumors attracts EPCs, thereby promoting vascular growth.^48^ In this intricate process, EPCs are recruited into TME and subsequently induced by chemokines released by tumor-associated ECs to infiltrate into the tumor vascular system, stimulating tumor angiogenesis.^49^ Furthermore, numerous oncogenic signals in TME drive EPCs to actively adhere to the tumor vascular system through integrin-mediated mechanisms (Fig. 1).^50^ SDF-1 plays a regulatory role in the tube-forming and adhesion of EPCs. In the hypoxic TME, SDF-1 triggers the engraftment of EPCs into the tumor vasculature and enhances their differentiation. Simultaneously, ECs derived from these EPCs exhibit heightened proliferative capabilities.^51^ On the other hand, EPCs were able to respond to different mechanical stimulations. Under 15 dyn/cm^2^ shear stress, the expression of C-X-C motif chemokine receptor 4 (CXCR4) was up-regulated and SDF-1-mediated Janus kinase (JAK)-2 phosphorylation was enhanced, which enhanced the homing, adhesion, and re-endothelialization functions of EPCs.^52^ In pathological conditions, the change of shape and structure of vascular endothelium subjected to oscillation shear force (OSS). In this mechanical environment, EPCs secrete increased levels of inflammatory proteins and begin to differentiate into mesenchymal cells, accelerating the pathological remodeling of blood vessels.^53^^,^^54^ Under the influence of OSS, OSS elevated the production of reactive oxygen species (ROS) and triggered phosphorylation via protein kinase Cζ (PKCζ), while concomitantly downregulating the expression of P53, thereby facilitating the differentiation of EPCs into mesenchymal cells. Exosomal circ-1199 also promoted the transformation of EPCs into mesenchymal cells by up-regulating the expression of high mobility group A2 (HMGA2).^53^^,^^55^ At the same time, HMGA2 is highly expressed in a variety of tumor tissues, and it promotes tumor growth, epithelial–mesenchymal transition, metastasis, and invasion.^56^ The performance of EPCs in the pathological vascular microenvironment may not only promote the pathological remodeling of blood vessels but also stimulate the further proliferation, metastasis, and invasion of tumor cells.

Pericytes, the stromal cells residing within the vascular system, exert a crucial role in vascular development and the maintenance of vascular function (Fig. 1). Diminished pericytes within the tumor vasculature cause heightened vascular permeability and an elevation in osmotic pressure within the intra-tumoral or mesenchymal regions. Consequently, this vascular dysregulation leads to a compromised blood flow in the vascular system and a decline in micro-ossvessel density. Concurrently, the reduced barrier function of the tumor vasculature is attributed to the inadequate coverage of pericytes, which further enhances tumor metastasis. Such promulgation of metastatic spread has been strongly correlated with an unfavorable prognosis in breast cancer.^9^ In single-cell sequencing experiments to analyze different pericyte subsets, tumor tissue-associated pericytes and normal tissue pericytes show great heterogeneity. TCF21 is highly expressed by a subset of tumor-associated pericytes, which promotes the degradation of the perivascular basement membrane and causes liver metastasis of colorectal cancer. The mechanism is related to the loss of integrin α5, which leads to the reduction of TCF21 promoter methylation and the increase of TCF21 expression.^57^ In tumor vascular remodeling, pericyte-expressed hexokinase 2 (HK2) impairs vascular support by activating ROCK2-MLC2, while inhibition of HK2 activity improves vascular function and enhances the delivery of chemotherapy drugs.^58^

Vascular smooth muscle cells (VSMCs) constitute a crucial component of the tunica media within blood vessels, playing a pivotal role in the contraction and relaxation of vascular tissues. However, within the TME, VSMCs frequently display aberrant morphological features. Simultaneously, the TME induces a phenotypic transition of VSMCs, shifting from a contractile state to a secretory and proliferative phenotype.^59^ This phenotypic transformation of VSMCs is often linked with alterations in Notch signaling.^60^ Regulator of G protein signaling 5 (RGS5) is prominently expressed in VSMCs and serves to inhibit vascular inflammation and remodeling. However, within the TME, RGS5 derived from tumors loses its beneficial function, inducing a shift in VSMCs towards a proinflammatory phenotype.^61^ On the one hand, abnormal shear stress in TME can directly stimulate VSMCs, promote their proliferation and migration ability, and lead to the remodeling of blood vessels. On the other hand, OSS-treated VSMCS can receive microRNA signals generated by ECs, which promote VSMC proliferation.^62^ The abnormal fluid dynamic signals in TME stimulate ECs to release micrornas, which may further lead to the dysfunction of VSMCs, and further lead to the pathological remodeling of blood vessels.

### Tumor vasculature influences TME

In tumor development, there exists a reciprocal relationship between TME and the tumor vascular system. Abnormalities in the vascular architecture of tumors result in increased tumor vascular permeability and facilitate tumor evasion.^63^ The heightened expression of insulin-like growth factor 1 (IGF-1) in ECs within the tumor vascular niche induces elevated expression of the IGF-1 receptor in adjacent cancer cells. This confers resistance to chemotherapy in cancer cells and enhances the invasiveness of tumors in close proximity to the vasculature.^64^ During the early stages of tumorigenesis, angiogenesis tends to be relatively inactive. However, as cancer cells proliferate, an abundance of angiogenic factors is secreted, prompting the induction of angiogenesis.^65^ Subsequently, the vascular system undergoes aberrant changes, driven by tumor-derived VEGF. This stimulus leads to abnormal outgrowth and permanent alterations in the phenotype of ECs within TME.^66^ Compared with normal blood vessels, the newly generated blood vessels in TME exhibit reduced efficiency in blood perfusion, resulting in tumor hypoxia.^43^ Tumor-associated ECs play a significant role in various processes including angiogenesis, tumorigenesis, immunosuppression, and chemoresistance through the secretion of multiple factors such as nitric oxide (NO), placental growth factor, angiopoietin 2 (ANGPT2), VEGFA, FGF2, and platelet-derived growth factor (PDGF).^67^ Tumor-associated ECs are also capable of recruiting TAMs via VEGF and C–C motif chemokine ligand 2 (CCL2), which further contribute to the promotion of angiogenesis within TME.^68^ Moreover, tumor-associated ECs express programmed cell death ligand 1 (PD-L1), a protein that binds to programmed death-1 (PD-1) receptors present in T-cells, consequently inhibiting the anti-cancer activity of T-cells.^69^ Therefore, an effective strategy against tumors involves blocking the expression of these aberrant proteins and restoring the normal vascular ecological niche.^64^

### Tumors respond to fluid shear forces within TME

FSS refers to the frictional force parallel to the flow surface exerted by viscous fluids. It stands as a pivotal mechanical factor within TME. The proliferation and apoptosis of tumors are intricately regulated by these fluid shear forces. Moreover, tumor-associated cellular factors produced within this microenvironment confer corresponding anti-shear properties to tumor cells, thereby promoting their adhesion to ECs and facilitating directed migration.^70^ In addition to its effects on tumor cells, fluid mechanics impact ECs, influencing their proliferation, tight junction integrity, and remodeling processes.^71^ Variations in FSS conditions similarly affect the proliferation and differentiation of EPCs.^53^ Dysregulation of fluid mechanical homeostasis within TME leads to aberrant release of chemotactic factors, hypoxia-inducible factors, and growth factors, thereby exacerbating tumor metastasis.^31^^,^^72^ The fluidic microenvironment of tumors is a complex mechanical milieu where tumor vessels transport nutrients to tumor tissues, while tumor cells and associated cytokines flow through the interstitial fluid toward lymphatic vessels. Pressure differentials between tumor vessels, tumor stroma, and lymphatic vessels constitute the fluid dynamics microenvironment of tumors.^73^^,^^74^ Early research suggests that tumor metastasis via the circulatory system is inefficient and spatially selective, potentially influenced by the TME.^75^ FSS emerges as a critical mechanical determinant shaping tumor behavior, with distinct FSS exerting significantly varied effects on tumor dynamics.^76^

### Cyclic fluid shear stress regulates cancer cell function

FSS can induce G2/M cell cycle arrest in cancer cells. In osteosarcoma cells, applying 12 dyn/cm^2^ shear stress activates integrin alpha v beta 3/1 (αvβ3/1), leading to Smad1/5 phosphorylation, which induces cell cycle arrest and differentiation inhibition.^77^ Simultaneously, Smad5 phosphorylation down-regulates the transcriptional activity of runt-related transcription factor 2 (Runx2).^77^ Within the tumor vascular microenvironment, high expression of MMP-9 promotes pathological angiogenesis, regulated upstream by Runx2, which when transcriptionally inactive may weaken MMP-9 expression, thus inhibiting pathological angiogenesis and maintaining normal blood shear stress.^78^^,^^79^ Laminar shear stress in normal vessels, acting on mechanosensitive bone morphogenetic protein (BMP) receptors I and II, activates Smad1/5 to promote P38 MAPK phosphorylation, enhancing levels of cystathionine γ-lyase (CGL) and light chain 3B II (LC3B-II) proteins, thereby inducing autophagy and apoptosis in various cancer cells.^80^ Conversely, oscillatory shear stress in tumor vessel pathology lacks these effects.^80^ Under the presence of tumor necrosis factor-related apoptosis-inducing ligand (TRAIL), FSS facilitates TRAIL binding to death receptors TRAIL-R1 and TRAIL-R2 on cell surfaces, activating CGL and initiating a caspase cascade, promoting apoptosis in colon and prostate cancer cells.^81^ β-catenin is closely linked to cell cycle regulation, apoptosis, and angiogenesis.^82^ β-catenin expression is regulated by shear stress levels; high FSS levels enhance mRNA expression of β-catenin, Bmi1, and c-myc. Extended exposure to lower shear stress indeed reduces CTC viability, yet increased shear stress levels correlate with heightened CTC activity.^83^ Similarly, short-term low-level laminar shear stress (2 dyn/cm^2^, 1 h or 2 h) induces Bcl-2 protein expression and increases focal adhesion kinase (FAK) phosphorylation through caveolin-1 (Cav-1) interaction with integrin β1, while lowering apoptosis-related proteins Bax and caspase-3 levels.^84^ Under such mechanical conditions, laminar shear stress also promotes Cav-1 dissociation from Fas, inhibits Fas dimerization, and suppresses death-inducing signaling complex (DISC) assembly and caspase-8 expression, thus hindering CTC apoptosis.^84^ CTCs' behavior varies significantly under different mechanical conditions, indicating a preference for specific shear stress intensities and durations. Relative to low FSS at venous levels (5 dyn/cm^2^), high FSS at arterial levels (15–30 dyn/cm^2^) can stimulate increased reactive oxygen species in CTCs, leading to mitochondrial dysfunction and promoting apoptosis. Simultaneously, elevated levels of FSS synergize with docetaxel and cisplatin to further elevate reactive oxygen species levels in cancer cells, enhancing their anti-cancer effects.^85^ Conversely, low-level laminar shear stress (2 dyn/cm^2^) treatment reduces the release of mitochondrial cytochrome c, resulting in higher mitochondrial depolarization potential in CTCs and reducing apoptotic events.^84^ In addition to regulating cellular activity, FSS also plays a role in cell adhesion. In prostate cancer, low levels of FSS enhance the adhesion of prostate cancer cells to vascular endothelium under the influence of E-selectin/SDF-1.^86^

### Circulating tumor resistance to fluid shear stress

Many studies currently indicate that physiological FSS can disrupt cancer cells significantly.^80^ However, the main cause of secondary tumor formation remains the circulation and metastasis of tumors.^87^ CTCs surviving FSS and immune killing exhibit enhanced stemness and invasiveness.^88^ Additionally, tumor resistance to FSS damage is associated with the activation of oncogenes like myc, phosphoinositide 3-kinase (PI3K), and H-ras. Mechanical forces such as shear stress induce cellular membrane damage.^89^ Loading of FSS on tumor cells confers them with increased resistance to shear stress, a process dependent on extracellular calcium ion concentration. Targeted inhibition of RhoA kinase (ROCK), which regulates actin dynamics, can reverse this process.^89^ Pulsatile FSS can promote apoptosis and necrosis of CTCs, although tumor cells show higher resistance compared with normal cells.^90^ Nuclear envelope proteins A/C (A/C) play a crucial role in transmitting mechanical forces from the cytoskeleton to the nucleus. A/C enables CTCs to resist FSS, and reducing A/C levels increases apoptosis and necrosis of CTCs.^90^ The distinct mechanical microenvironment is a critical difference between CTCs and primary TME. CTC DNA from breast cancer patients, unlike primary tumors, can withstand chemotherapy-induced damage.^91^ Apart from solid mechanics, this drug resistance process likely relates to FSS stimulation. Yes-associated protein 1 (YAP1) is a mechanical force-sensitive protein that is inhibited under classical shear stress in blood vessels to inhibit cell motility. However, FSS (0.05 dyn/cm^2^) in the lymphatic vessels up-regulates YAP1 via ROCK through the LIMK-cofilin pathway to promote prostate cancer metastasis.^92^ Unfortunately, abnormal tumor vasculature often leads to high interstitial pressure, hypoxia, and increased HIF-1α expression, enhancing shear stress levels in lymphatics.^93^ This microenvironment promotes the proliferation of lymphatic endothelium and CTC migration from vessels to lymphatics.^9^^,^^93^

Moreover, under FSS, CTCs acquire physiological functions similar to ECs. For instance, in ovarian cancer OVCAR-3 cells, FSS up-regulates calcium-binding mitochondrial carrier proteins Aralar1 and 2 involved in cholesterol biosynthesis pathways. Similar to ECs responding to shear stress, this stimulation regulates cholesterol metabolism, mitochondrial oxidative phosphorylation, calcium ion transport, and ATP generation. This suggests that CTCs selected under shear stress, with enhanced stemness, may differentiate into endothelial-like cells or acquire similar physiological functions.^94^ Similar tumor–endothelial transition phenomena occur in melanoma. Tumor cells generate a perivascular system on organ surfaces via intercellular adhesion molecule-1 (ICAM-1), forming a vascular niche similar to ECs. This tumor–endothelial transition may further deteriorate the tumor vascular microenvironment, including tumor vascular mechanical conditions.^95^ Overall, these findings underscore the complex interplay between mechanical forces like FSS and tumor biology, influencing metastasis and therapeutic responses in cancer.

### Role and mechanism of natural medicines on tumor angiogenesis

There are two main treatment strategies for the tumor vasculature system. One targets abnormal vessel regeneration, utilizing natural agents that target VEGF and other angiogenic factors to reduce pathological vessel formation.^10^^,^^96^ This approach aims to decrease tumor nutrient supply and the number of vessels generating abnormal FSS, thereby controlling tumor metastasis and invasion via the vascular system. The other strategy focuses on normalizing existing vessels, improving their permeability, and maintaining their geometric integrity. This prevents abnormal interstitial flow caused by increased vessel permeability and ensures normal FSS.^97^^,^^98^

### Inhibition of angiogenesis

#### Phenolic

Resveratrol is a naturally occurring non-flavonoid polyphenol derived from plants. In HUVECs, resveratrol demonstrated the ability to hinder the VEGF-induced tyrosine phosphorylation of vascular endothelial-calmodulin and its associated chaperone, β-catenin, consequently impeding angiogenesis.^10^ In myeloma, resveratrol effectively suppresses the tube-forming capacity of HUVECs through the down-regulation of pivotal angiogenic factors such as VEGF, bFGF, MMP-2, and MMP-9.^96^ Furthermore, resveratrol indirectly inhibits the proliferation and migration of ECs by attenuating the secretion of VEGF and IL-8/CXCL8 by human peritoneal mesothelial cells.^99^ In hepatocellular carcinoma, resveratrol exerts its anti-angiogenic effects by reducing the expression of VEGF and IL-2 within tumors, achieved through the inhibition of glioma-1 (Gli-1).^100^ Notably, resveratrol also showed the capability to impede adenosine diphosphate-induced platelet activation and VEGF release. This action is mediated by an increase in cyclic guanosine monophosphate production and the subsequent phosphorylation of vasodilator-stimulated phosphoprotein (VASP) (Table 1).^101^

**Table 1:** Action factors of natural medicines on cancer.

Natural medicine	Action factors	Related cancer	Reference
Resveratrol	VEGF, IL-6, bFGF, MMP-2, MMP-9, IL-8, CXCL8, IL-2, Gli-1, VASP, Ca^2+^	Colon cancer, multiple myeloma, liver cancer	10,96,99, 100, 101,141
Curcumin	FGF, PDGF, IL-8, VEGF, MMP-2, MMP-9, NF-κB, IL-1	Rectal cancer, lung cancer, breast cancer, lymphoma	102,103,142, 143, 144
Quercetin	VEGFR-2, HIF-1α, NFAT, HSP2, COX1, Bcl-2, Bax	Breast cancer, prostate cancer, lung cancer, glioma, ovarian cancer	105, 106, 107,145,146
Honokiol	VEGF, HO-1, GPX4, EGFR, CD133, AMPK, Sirt3, Notch-2	Kidney cancer, colon cancer, glioma, liver cancer, melanoma	109,147, 148, 149, 150
Hesperidin	VEGF, COX-2, PinX1, ROS, NFATc3	Kidney cancer, lung cancer, esophagus cancer, stomach cancer, breast cancer	107,112,151, 152, 153
Psammaplin A	APN, DOT1L	Breast cancer	114,154
Ginkgolide	PAFR, NF-кB, c-Met, STAT3	Pancreatic cancer, liver cancer, lung cancer, ovarian cancer	155, 156, 157, 158
Ursolic acid	VEGF, NF-κB, STAT3, Akt, SHH, bFGF, CDK1, CDK4, MMP-2, AI-1, TIMP-2, ERK, JNK, p38, p62	Prostate cancer, colon cancer, breast cancer, rectal cancer, oral squamous cell carcinoma	117,118,159, 160, 161
Artesunate	COX-2, PGE2, VEGFR2, EGFR, c-MET, Src, FAK, PGE2, FOXP3, Beclin1, DNMT3b	Bladder cancer, kidney cancer, cervical cancer, breast cancer, prostate cancer	162, 163, 164, 165, 166
Penduliflaworosin	VEGFR2		121
Paclitaxel	bFGF, MMP-2, vimentin, MMP9, VEGF, TGFA, FGF, IL-6	Breast cancer, head and neck squamous cell carcinoma, lung cancer	11,125,167,168
Colchicine	Bax, Bcl-2, PI3K, Akt, mTOR, Cytochrome c, caspase-3	Stomach cancer, breast cancer, lung cancer, colon cancer,	169, 170, 171, 172
Combretastatin	HIF-1α, PD-L1, BubR1, MPM-8301, MPM-2	Liver cancer, ovarian cancer, bladder cancer, breast cancer	173, 174, 175, 176
Vincristine	CCNB1, AURKA, NFκB, JNK, Bax, Bcl-2, Bcl-xL, MMP-2, MMP-9	Lung cancer, prostate cancer, leukemia, liver cancer	177, 178, 179, 180
Camptothecin	Nrf2, VEGFR, p21, CDK2, Cyclin A, Bax, caspase-3, Bcl-2, DNA TopI, AKT, MEK, ERK, JNK2, p38, MAPK, CCNA2, CDK2, MYBL2	Liver cancer, esophageal squamous cell carcinoma, bladder cancer, stomach cancer, colon cancer	134,181, 182, 183, 184
Berberine	MMP-2, μ-PA, VEGF, Bax, JNK, Beclin1, Bcl-2, AKT, mTORC1, cyclin B1, cdc2, cdc25c, PI3K, mTOR	Breast cancer, leukemia, colorectal cancer, stomach cancer	133,185, 186, 187, 188
Crustal oligosaccharides	VEGF, caspase-3, Bax, Bcl-2, ROS, Nrf2	Lung cancer, kidney cancer, colon cancer, stomach cancer, cervical cancer	137,189, 190, 191, 192, 193
Shenmai injection	VEGF, FGF, PAI-10, PD-1, GPER1, Foxp3	Colorectal cancer, lung cancer, thyroid cancer	98,194,195

Curcumin, a phenolic compound derived from the rhizome of turmeric, is a crucial constituent of the curcumin family. It exhibits anti-tumor properties by inhibiting the growth of numerous tumors and impeding vascular smooth muscle migration, proliferation, and collagen synthesis.^102^ In the context of pancreatic and ovarian tumors, curcumin inhibits VEGF, thereby exerting angiogenesis inhibition similar to that of bevacizumab.^102^ Moreover, in colorectal cancer, curcumin effectively hinders tumor angiogenesis by blocking the JAK/STAT3/IL-8 signaling pathway (Table 1).^103^

Quercetin, a flavonoid compound with polyphenolic properties, is abundantly present in various foods and plants. It possesses notable anti-inflammatory, antioxidant, and anti-tumor properties.^104^ Quercetin exerts its effects by inhibiting the phosphorylation of VEGFR2 on the surface of ECs, activating downstream molecules including protein kinase B (AKT), mammalian target of rapamycin (mTOR), and p70S6K, and impeding the proliferation, migration, and tube formation of ECs. This substantiates its anti-tumor angiogenic activity both *in vivo* and *in vitro*.^105^ Furthermore, in the case of tamoxifen-resistant breast cancer, quercetin demonstrates inhibitory effects on Pin, a crucial factor for angiogenesis, as well as on HIF-1α and c-Jun/AP-1, key transcription factors responsible for VEGF gene transcription.^106^ Moreover, traditional Chinese medicine formulations containing quercetin as the primary active ingredient are capable of down-regulating nuclear factor of activated T-cells cytoplasmic 3 (NFATc3) through the activation of the nuclear factor family of T-cells (NFAT) signaling pathway. This mechanism has been found to reduce blood vessel density in breast cancer (Table 1).^107^

Honokiol acts on the mitogen-activated protein kinase (MAPK)/mTOR signaling pathway, leading to a reduction in the expression of proteins such as VEGFR2 and platelet endothelial cell adhesion molecule (PECAM). It enables honokiol to exert potent anti-angiogenic effects.^108^ In renal tumors, honokiol counteracts the CsA-induced elevation of VEGF expression, thereby attenuating angiogenesis and inhibiting renal tumor growth.^109^ Honokiol derivatives have been shown to disrupt the F-actin cytoskeleton, effectively inhibiting pseudopod formation and reducing the invasive migration of ECs. Additionally, these derivatives inhibit the expression and phosphorylation of extracellular signal-regulated kinase (ERK). Consequently, they also hinder tube formation by HUVECs and reduce neovascularization in zebrafish models (Table 1).^110^

Hesperidin belongs to the class of polyphenolic compounds found in citrus fruits and possesses significant anti-cancer potential.^111^ Hesperidin effectively counteracts the up-regulation of VEGF induced by renal cancer inducers, namely, diethyl nitrosamine (DEN) and ferric nitrilotriacetate (Fe-NAT). This action results in the inhibition of angiogenesis, consequently conferring a preventive effect against renal cancer.^112^ Furthermore, hesperidin exhibits the capability to selectively target a diverse array of proteins implicated in tumor angiogenesis and metastasis, including cyclooxygenase-2 (COX-2), MMP-2, and MMP-9 (Table 1).^113^

Psammaplin A, derived from marine biological sponges, is a phenolic natural compound renowned for its anti-tumor effects. Throughout tumor development, mammalian aminopeptidase N (APN) plays a crucial role in tumor invasion and angiogenesis. Psammaplin A exerts its inhibitory effects on ECs using non-competitive inhibition of APN, effectively suppressing their proliferation. Furthermore, psammaplin A demonstrates the ability to impede bFGF-induced angiogenesis (Table 1).^114^

#### Terpene

In colitis-associated cancer, platelet-activating factor (PAF) plays a role in tumor angiogenesis. Ginkgolide B hampers the translation of VEGF, inhibits PAF signaling, and enhances the activity of PAF acetylhydrolase (PAF-AH), thereby impeding tumor angiogenesis (Table 1).^115^ Ursolic acid, a natural oleanane triterpenoid derived from various plants, exhibits anti-cancer properties by inhibiting tumor proliferation, metastasis, and angiogenesis.^116^ In prostate cancer, ursolic acid suppresses the expression of VEGF through nuclear factor kappa B (NF-κB) and STAT3 pathways, resulting in anti-angiogenic and pro-apoptotic effects.^117^ Moreover, in colon cancer, ursolic acid inhibits the activation of STAT3, Akt, and sonic hedgehog (SHH) pathways, down-regulates the expression of pro-angiogenic factors VEGF and bFGF, and impedes the proliferation and migration of ECs, thereby exerting an inhibitory effect on tumor angiogenesis (Table 1).^118^ Artesunate, a derivative of artemisinin isolated from the Chinese herb *Artemisia annua*, belongs to the sesquiterpene lactone class of compounds.^119^ In chronic granulocytic leukemia, elevated levels of VEGF are observed. Artesunate inhibits the expression of VEGF, leading to reduced vascular migration (Table 1).^120^ Penduliflaworosin, derived from the traditional Chinese medicine *Croton crassifolius Geiseler*, is a diterpenoid compound.^121^ It attenuates the activity of VEGFR2 tyrosine kinase and interferes with downstream phosphorylation of AKT, mTOR, FAK, and ERK, which hinders the proliferation, invasion, migration, and tube formation of HUVECs. Additionally, penduliflaworosin blocks VEGF-induced aortic ring sprouting and angiogenesis in both rats and mice (Table 1).^121^

#### Alkaloid

Paclitaxel, a natural alkaloid derived from the bark and needles of *Taxus brevifolia*, exerts its anti-cancer effects through several mechanisms. It primarily inhibits cancer cell growth by binding to the β-subunit of microtubules, thereby impeding mitosis and disrupting cell cycle progression.^122^ Additionally, paclitaxel exerts inhibitory effects on ECs by promoting their apoptosis and attenuating their migration and invasion.^123^ Furthermore, paclitaxel modulates the response of ECs to tumor-derived angiogenic factors. It hinders the action of the chemokine bFGF and reduces the production of MMP-2, ultimately leading to a pronounced anti-angiogenic effect.^11^ In addition to its impact on ECs, paclitaxel also inhibits the proliferation of EPCs and enhances their apoptosis.^124^ In breast cancer, a synergistic therapeutic strategy entails the co-administration of paclitaxel with a Wnt signaling inhibitor, XAV939. This combinatorial therapeutic approach leads to the down-regulation of vimentin and MMP9, the up-regulation of E-calmodulin, which acts as an inhibitor of epithelial–mesenchymal transition, and a reduction in mRNA levels associated with vascular markers. These molecular alterations collectively culminate in the inhibition of angiogenesis within TME (Table 1).^125^

Colchicine, a naturally occurring alkaloid extracted from the Colchicum genus, demonstrates potent anti-tumor properties. It exerts its effects by binding to microtubulin, influencing the assembly of the mitotic spindle, and ultimately resulting in cell cycle arrest and apoptosis.^126^ Recent research has unveiled an additional mechanism of colchicine, where it depolymerizes actin. Colchicine counteracts tumor vascularization by disrupting actin polymerization, leading to a significant reduction in angiogenesis. In combination with other chemotherapeutic agents, colchicine mitigates adverse effects such as the dissemination of metastases, a consequence of the formation of new tumor vessels following chemotherapy.^127^ Similarly, like paclitaxel and colchicine, combretastatin is derived from the African dwarf willow tree. Combretastatin exerts anti-tumor effects by inhibiting the polymerization of microtubule proteins, resulting in microtubule destabilization and subsequent inhibition of cell proliferation. Furthermore, combretastatin serves as a vascular-disrupting agent (Table 1).^128^

Lin et al^129^ observed that low doses of vincristine disrupted the formation of endothelial tip cells and led to a reduction in vascular branching. Conversely, high doses of vincristine resulted in a decrease in the acetylation levels of ECs' microtubule proteins, impairing the formation of lumens (Table 1).

Camptothecin, derived from the bark of *Camptotheca acuminata*, is a traditional Chinese medicine renowned for its anti-cancer properties.^130^ Derivatives of camptothecin, such as irinotecan, have demonstrated efficacy in the treatment of cervical, lung, and ovarian cancers.^131^ In liver cancer, camptothecin has been observed to modulate the epithelial–mesenchymal transition by influencing the expression of key markers. Specifically, it enhances the expression of the epithelial marker E-cadherin while concurrently suppressing the expression of the mesenchymal marker N-calmodulin. This regulatory action is attributed to the inhibition of nuclear factor erythroid 2-related factor 2 (Nrf2).^12^ Furthermore, camptothecin exerts anti-angiogenic effects by reducing the expression of vascular endothelial VEGFR and diminishing the number of blood vessels in hepatocellular carcinoma. The down-regulation of VEGFR is linked to the inhibition of Nrf2 (Table 1).^12^

Berberine is a benzyltetraisoquinoline alkaloid, a naturally occurring plant compound with antibacterial properties. Berberine exhibits inhibitory effects on a wide spectrum of cancers, including breast, colon, pancreatic, gastric, liver, oral, and bone cancer and glioblastoma.^132^ During the development of tumor vasculature, berberine reduces the expression of invasive enzymes MMP-2 and μ-PA and down-regulates VEGF in HUVECs. The modulation impedes angiogenesis and disrupts the development of vasculature in zebrafish.^133^ Furthermore, berberine inhibits the activation of MMP-1 and MMP-9 induced by reactive oxygen species, thereby suppressing angiogenesis within tumors (Table 1).^134^

### Promotion of blood vessels' normalization

Crustal oligosaccharides, derived from the exoskeleton of arthropod insects and the cell walls of fungi, exhibit anti-inflammatory and anti-tumor properties.^135^ Moreover, crustal oligosaccharides and their derivatives have anti-angiogenic effects, inhibiting the proliferation, migration, and tube formation of ECs.^13^^,^^14^ Chitosan derivatives reduce the expression or secretion of VEGF, inhibiting the sprouting of small capillaries and the growth of tumor blood vessels (Table 1).^136^ Carboxymethyl chitosan has demonstrated the capability to diminish the levels of VEGF and MMP-9, thereby manifesting anti-tumor efficacy.^137^ Partially acetylated chitosan likewise exhibits the ability to attenuate MMP-9 activity, mitigate EC permeability, impede vascular infiltration by liver cancer cells, and reduce the adhesion of cancer cells to ECs.^97^ Shengmai injection (Table 1) (made from purified ginseng, *Radix Ophiopogonis*, and *Schisandra chinensis*) is primarily composed of red and yellow ginseng, with ginseng saponin as the main active ingredient. Shengmai injection can increase pericyte coverage around tumor blood vessels.^98^ When combined with 5-fluorouracil chemotherapy, Shengmai injection reduces micro-vessel density, balances pro-angiogenic and inhibitory angiogenic factor signaling, increases perivascular cell coverage, and promotes the formation of mature vessels.^98^ Additionally, the anti-tumor effect of paclitaxel is enhanced when used in combination with Shengmai injection.^138^ The more action factors of the above natural drugs and the main types of cancer treated by these drugs are shown in Table 1.

## Summary and outlook

To facilitate the rapid proliferation of cells, tumors develop a dense and irregular vascular network. However, the vascular microenvironment within tumors is abnormal, characterized by an abundance of immature vessels that exhibit excessive permeability and poor perfusion efficiency. As a result, cancer cells can disseminate and metastasize, and chemotherapeutic agents are not effectively delivered. Recent studies have shown that tumor vascular cells and normal controls are very different.^57^ In the study of tumor blood vessels, it may be necessary to use more primary culture cell extracted from tumor tissues to better simulate the state of these vessel-associated cells in TME. However, the content of non-tumor cell components from tumor tissue is relatively small, and the extraction of primary culture cells is slightly difficult. In the anti-angiogenic approach, natural medicines targeting the vascular microenvironment primarily inhibit EC-mediated vascular outgrowth and angiogenesis by modulating cytokines such as VEGF, PDGF, and MMPs, the occurrence of epithelial–mesenchymal transition, and the cytoskeletal function of ECs. However, the use of VEGF inhibitors may increase the risk of hypertension, epistaxis, and venous thrombosis.^139^^,^^140^ In terms of improving the tumor vascular microenvironment, natural drugs have fewer side effects compared with chemotherapy drugs. At the same time, natural drugs can enhance the anti-tumor effect of chemotherapy drugs and reduce their adverse reactions by maintaining normal vascular function and promoting chemotherapy drugs to enter the depth of the tumor.^98^^,^^138^ Tumors exhibit heterogeneity, and different natural medicines have distinct targets for modulating the tumor vascular microenvironment, resulting in variable therapeutic effects among different tumor patients. However, there are still some limitations in the research of natural drugs, such as the unclear active ingredients and the unclear mechanism of action. The active components of extraction of natural medicine and its promoting vascular normalization can more accurately target different tumors of different targets. Therefore, it is possible to screen more specific therapeutic options based on the characteristics of different tumors and drug targets. Promoting the normalization of the tumor vascular hydrodynamic microenvironment may be a key goal to improve the overall TME.

## CRediT authorship contribution statement

**Yirui Lu:** Writing – original draft. **Zhiliang Guo:** Writing – original draft. **Hong Li:** Supervision. **Jiao Wen:** Supervision. **Xiaoyun Zhang:** Conceptualization. **Xiumei Guan:** Writing – review & editing. **Xiaodong Cui:** Funding acquisition, Writing – review & editing. **Min Cheng:** Funding acquisition, Writing – review & editing.

## Funding

This study was supported by the Weifang Science and Technology Development Projects (Shandong, China) (No. 2023YX092).

## Conflict of interests

The authors have no competing interests to declare.
